# Unravelling the Photoprotection Properties of Garden Cress Sprout Extract

**DOI:** 10.3390/molecules26247631

**Published:** 2021-12-16

**Authors:** Temitope T. Abiola, Nazia Auckloo, Jack M. Woolley, Christophe Corre, Stéphane Poigny, Vasilios G. Stavros

**Affiliations:** 1 Department of Chemistry, University of Warwick, Coventry CV4 7AL, UK; temitope.abiola@warwick.ac.uk (T.T.A.); Nazia.Auckloo@warwick.ac.uk (N.A.); jack.woolley@warwick.ac.uk (J.M.W.); C.Corre@warwick.ac.uk (C.C.); 2 Warwick Integrative Synthetic Biology Centre and School of Life Sciences, University of Warwick, Coventry CV4 7AL, UK; 3 Mibelle Group Biochemistry, Mibelle AG, Bolimattstrasse 1, CH-5033 Buchs, Switzerland; stephane.poigny@mibellegroup.com

**Keywords:** sunscreen, UV filter, photoprotection, nature-inspired, photodynamics, photophysics, sinapoyl malate, plant sunscreen, photochemistry, ultrafast spectroscopy

## Abstract

Plants, as with humans, require photoprotection against the potentially damaging effects of overexposure to ultraviolet (UV) radiation. Previously, sinapoyl malate (SM) was identified as the photoprotective agent in thale cress. Here, we seek to identify the photoprotective agent in a similar plant, garden cress, which is currently used in the skincare product Detoxophane nc. To achieve this, we explore the photodynamics of both the garden cress sprout extract and Detoxophane nc with femtosecond transient electronic absorption spectroscopy. With the assistance of liquid chromatography-mass spectrometry, we determine that the main UV-absorbing compound in garden cress sprout extract is SM. Importantly, our studies reveal that the photoprotection properties of the SM in the garden cress sprout extract present in Detoxophane nc are not compromised by the formulation environment. The result suggests that Detoxophane nc containing the garden cress sprout extract may offer additional photoprotection to the end user in the form of a UV filter booster.

## 1. Introduction

**Figure 1:**
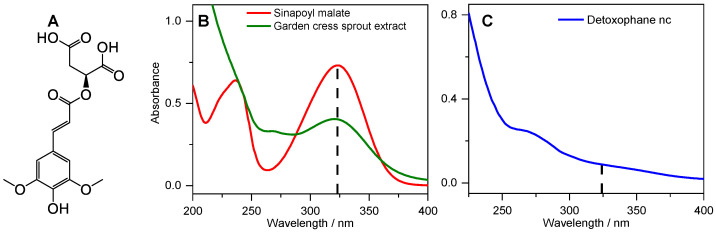
(**A**) Molecular structure of *trans*-sinapoyl malate. UV–Vis spectra of samples obtained for (**B**) 0.01 mg/mL *trans*-sinapoyl malate in water (red), 0.1 mg/mL of garden cress sprout extract in water (green), and (**C**) Detoxophane nc diluted at a ratio of 1:100 in water.

The need for adequate protection of human skin against high doses of ultraviolet (UV) radiation exposure from the Sun has continued to generate great interest, owing to the well-reported adverse effects of overexposure to UV radiation [1,2,3,4,5]. Currently, several sunscreen formulations are on the market, with active ingredients ranging from inorganic UV filters such as titanium dioxide (TiO_2_) and zinc oxide (ZnO) to organic UV filters, examples of which include avobenzone, oxybenzone, homosalate, and octocrylene, among many others [3,6]. Despite the many sunscreen formulations currently available, certain setbacks such as the potential toxicity to humans and the environment, as well as photo-instability, have resulted in the banning of some UV filters [7,8,9,10]. To this effect, sunscreen scientists have continued to search for safe and efficient UV filters, seeking inspiration from natural sources, including plants and microorganisms [11]. For example, in plants, sinapoyl malate (SM, see Figure 1A), found in the upper layer of Brassicaceous plants such as thale cress (*Arabidopsis thaliana*), has been reported to be the photoprotective agent [12,13]. In order to elucidate the photoprotection mechanism, the photodynamics of SM and its derivatives have also been studied [11,14,15,16,17,18,19]. Briefly, these studies have shown that following UV absorption, SM and its derivatives undergo an efficient and ultrafast energy relaxation mechanism, predominantly *trans*-to-*cis* photoisomerisation, which accounts for their long-term photostability and photoprotective nature. Furthermore, an additional important advantage of these plant-based UV filters is the broad UV absorption profile that covers the UVB (280–315 nm) region of the solar spectrum and extending to the UVA (315–400 nm), where there is a sparsity of efficient UV filters.

In this work, we examine the potential photoprotection properties in a nature-inspired active skincare ingredient currently on the market. Detoxophane nc, consisting of *Lepidium sativum* (garden cress) sprout extract (referred to as “cress sprout extract” henceforth), prepared in polar solubilisers and encapsulated with liposome (lipid vesicles), a versatile platform as a carrier for delivery of skincare products and drugs into the human body [20], has been developed by Mibelle Group Biochemistry for application as an anti-ageing skincare product [21]. This active ingredient helps to protect the skin cells against adverse environmental pollutants and other intrinsic reactive molecules (e.g., reactive oxygen species, ROS). However, since garden cress is closely related to thale cress, it is plausible to suggest that SM or other related derivatives could be responsible for the protection afforded by the plant. To this effect, we have focused our efforts on the photoprotection properties of both the cress sprout extract and Detoxophane nc through the use of transient electronic absorption spectroscopy (TEAS). This should enable us to access any potential effects of the complex surrounding environment on the photodynamics of both cress sprout extract and Detoxophane nc. The insights garnered may shed light on how the cress sprout extract can be potentially used as a UV filter booster in skin-care formulations. Indeed, our results reveal that SM is the predominant UVB–UVA filter in the cress sprout extract, thereby offering photoprotection to the plant. The photodynamics of both cress sprout extract and Detoxophane nc are near-identical and compare well with the previously reported photodynamics of SM. Crucially, this suggests that the photoprotection properties of the SM in the cress sprout extract present in Detoxophane nc are not compromised by the surrounding environment.

## 2. Results

### 2.1. Steady-State Spectroscopy

UV–Vis absorption spectra of the cress sprout extract and Detoxophane nc were measured in deionised water. As shown in Figure 1B, the UV absorption profile of the cress sprout extract displays a broad absorption band with an absorption maximum (λ_max_) in the UVA region (324 nm). The UV spectrum compares well with that of synthetic *trans*-SM in Figure 1B, having the same λ_max_ and a similar absorption profile. This is an indication that the UV-absorbing species in the cress sprout extract is either SM, a derivative, or a species with a similar absorption profile to SM. Furthermore, previous studies have reported that SM in nature occurs in the *trans*-isomer [17,22]. Hence, we might expect that SM in the cress sprout extract is in the same isomeric form. The agreement between the λ_max_ of synthetic *trans*-SM and cress sprout extract together with the results of the ^1^H NMR reported in the Supplementary Information Figures S1 and S2 supports the expectation that the *trans*-isomer of SM is the absorbing species; indeed, the coupling constants for H-3/H-4 are identical (16 Hz) and consistent with those expected for a *trans*-isomer of the SM derivative (ethyl sinapate) rather than a *cis*-isomer [17].

In contrast to the cress sprout extract, the absorption of Detoxophane nc (Figure 1C) is rather broad and almost featureless; we suggest this is due to the dilution of the cress sprout extract in Detoxophane nc and the scattering from other components within the formulation. In this case, the UV-absorbing species in the formulation would be hidden under the scattered spectrum from other components in the active ingredient.

Furthermore, the photostability of the cress sprout extract was explored in water as described in the methods section (see Section 4). The results presented in Supplementary Information Figure S3 revealed that the absorbing species demonstrates high photostability with only an 18% reduction in the absorbance at the absorption maximum (λ_max_) at a photostationary state (PSS) after 120 min of irradiation. This loss in absorbance is suggested to be a consequence of photoisomerisation, since the *cis*-isomer of SM derivatives have been previously reported to possess lower extinction coefficient [17,23]. In order to confirm the process of photoisomerisation in cress sprout extract leading to the formation of *cis*-isomer, samples of both SM and cress sprout extract were irradiated and the ^1^H NMR were obtained (see Supplementary Information Section S2 for detail [17]). The results presented in Supplementary Information Figures S4 and S5 confirm the formation of the *cis*-isomer of SM in both samples, with the coupling constants of the *cis*-isomer for H-3c/H-4c being identical for both samples (12 Hz) and consistent with those expected for a *cis*-isomer of SM derivative (ethyl sinapate) [17]. Furthermore, we have estimated the photoisomerisation quantum yield of the *cis*-isomer of SM from the ^1^H NMR spectrum after irradiation. The result showed that 10% of the starting *trans*-SM was converted to *cis*-SM following 2 h of irradiation.

### 2.2. Identification of the UV-Absorbing Species

**Figure 2:**
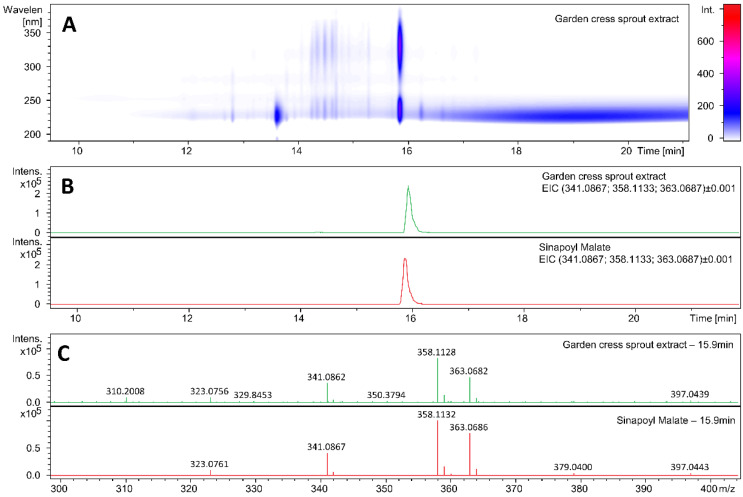
(**A**) UHPLC analysis of the garden cress sprout extract, highlighting compounds eluting at between 10 and 20 min and absorbing in the 210–390 nm range. (**B**) Extracted ion chromatogram of cress sprout (green) and sinapoyl malate standard (red) for *m/z* values calculated for [SM+H]^+^, SM ammonium adduct, and SM sodium adduct (**C**) Mass spectra for cress sprout and sinapoyl malate at retention time of 15.9 min.

To identify the molecule responsible for the UV absorption in cress sprout, ultra-high performance liquid chromatography–high-resolution mass spectrometry (UHPLC–HRMS) measurements were carried out. The retention time on the UHPLC column as well as the high-resolution mass spectra of the absorbing compound in the cress sprout were compared to those of synthetic *trans*-SM, as reported in Figure 2. Figure 2A shows the UHPLC analysis of the garden cress sprout extract, highlighting compounds eluting between 10 and 20 min and absorbing in the 210–390 nm range. The extracted ion chromatogram (EIC) *m/z* values calculated for protonated SM (341.0867; [C_15_H_16_O_9_+H]^+^), for the ammonium SM adduct (358.1133; [C_15_H_16_O_9_+NH_4_]^+^), and for the sodiated SM adduct (363.0687; [C_15_H_16_O_9_+Na]^+^) shown in Figure 2B revealed they were similarly retained on the UHPLC column, with a retention time ~15.9 min (see Supplementary Information Figure S6 for the UV chromatograms of the sample, standard, as well as the sample co-injected with the standard). The UV absorption spectrum of the cress sprout eluted at this time is shown in Supplementary Information Figure S7 and matches that of the SM reported in Figure 1, with a slight change in the absorption maximum, likely due to the difference in the solvent environment, i.e., 50:50 *v/v* water/methanol vs. water. The mass spectra at a retention time of 15.9 min., as reported in Figure 2C, also confirmed the presence of SM in the garden cress sprout extract. The same fingerprint was observed for the extract and the synthetic SM standard and molecular formulae corresponding to the ions detected in the extract matched with those of SM and SM adducts.

### 2.3. Transient Electronic Absorption Spectroscopy (TEAS)

**Figure 3:**
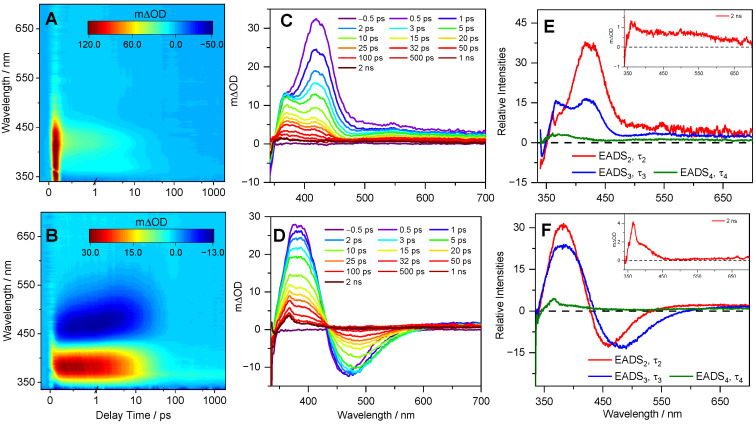
TEA spectra obtained for 0.1 g of cress sprout extract in 25 mL of (**A**) dioxane and (**B**) water, photoexcited at 330 nm, with spectra presented as false colour maps. The same data are presented as line plots of mΔOD vs. probe wavelength at selected pump–probe delay times in (**C**,**D**) for cress sprout extract in dioxane and water, respectively. (**E**,**F**) The evolution associated difference spectrum (EADS) for cress sprout extract in dioxane and water, respectively, produced by the fitting procedure. The inset (**E**,**F**) shows the transient absorption spectrum at the maximum available pump–probe delay of 2 ns.

We first consider the cress sprout extract. The transient electronic absorption (TEA) spectra measured following photoexcitation at 330 nm in a separate solution of dioxane and water are presented in Figure 3. We chose weakly (dioxane) and strongly (water) interacting solvents to investigate how the solvent–solute interaction influences the dynamics; this was also in keeping with previously reported studies for sinapate ester derivatives. Consequently, the assignment of the observed features herein closely follows the literature [14,17,19,22,23,24,25]. For the cress sprout extract in dioxane (Figure 3A), the TEA spectra are dominated by three features. The first is a negative signal observed below ~350 nm (more visible in the line plots in Figure 3C), which is assigned to a ground state bleach (GSB) of the photoexcited SM molecules following comparison with the static UV absorption spectrum. This feature persists throughout the maximum pump–probe delay time (Δ*t* = 2 ns) of our experimental setup. The second is an intense positive signal peaking at ~400 nm, which predominantly decays to baseline within ~50 ps; a small component persists beyond 2 ns, which is discussed below. Finally, there is a second broad and weak absorption spanning the spectral region of ~500–700 nm. The two positive absorption features have been assigned previously in sinapate esters and similar systems to the excited state absorption (ESA) of the first singlet excited state (i.e., S*_n_* ← 1^1^ππ*), since initial photoexcitation promotes a 1^1^ππ* state [14,17,19]. The TEA spectra of the cress sprout extract in water shown in Figure 3B are also dominated by the three features seen in dioxane. In addition, a negative signal centered at ~470 nm is clearly present and assigned to stimulated emission (SE). This feature decays back to baseline by ~50 ps, a similar timescale at which the ESA at ~400 nm decays. As a comparison, the TEA spectra of SM in water are reported in Figure S8. The data revealed the same spectral profile and a comparable time constant (see Table 1) as those obtained from cress sprout extract in water.

**Figure 4:**
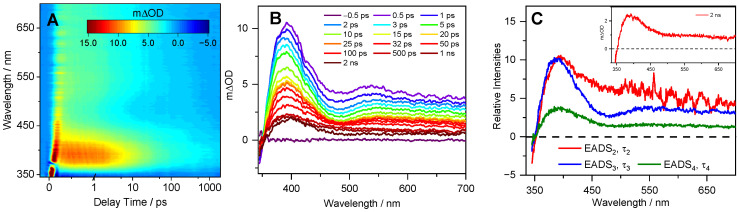
TEA spectra obtained for the bulk solution of Detoxophane nc photoexcited at 330 nm, shown as a false colour map (**A**). The same data are presented as a line plot of the mΔOD vs. probe wavelength at selected pump–probe delay times in (**B**). The EADS is shown in (**C**), with the 2 ns transients presented as an inset.

Furthermore, the TEA spectra of the Detoxophane nc is shown in Figure 4. Briefly, the TEA spectra consist of three features, a GSB observed below ~350 nm, ESA centered at ~400 nm, and a second broad ESA that spans ~500–700 nm. We add that the TEA spectra in Detoxophane nc more closely resemble the cress sprout extract in a weakly interacting solvent (dioxane) as compared to a strongly interacting solvent (H_2_O), as evidenced by the absence of the SE feature. We discuss this in more detail below.

Quantitative insight into the photodynamical process observed in the TEA spectra of both cress sprout extract and Detoxophane nc is obtained by employing a global sequential ($A\overset{\tau_{1}}{\to }B\overset{\tau_{2}}{\to }C\overset{\tau_{3}}{\to }D\ldots$) decay model, implemented through the Glotaran software package [26,27]. The extracted time constants are reported in Table 1, while the quoted errors are those returned by the fitting software to 2 standard error, although the quality of the fits are better evaluated by inspecting the associated residuals reported in Supplementary Information Figure S9. When the error returned by the fitting package was shorter than the instrument response time, the error was quoted as half the instrument response, as determined via the solvent-only transients presented in the Supplementary Information Figure S10 [28].

## 3. Discussion

We now discuss the implications of our results with respect to photoprotection, drawing on different aspects of the experimental studies. The UV–Vis and UHPLC–HRMS spectra suggest that the absorbing species in the cress sprout extract is SM. In Detoxophane nc bulk solution, the UV absorption is featureless due to the lower concentration of the absorbing species and scattering effect of other components of the formulation.

With regards to the ultrafast photodynamics of the cress sprout extract and the Detoxophane nc solution, we draw on our experimental results and previous studies, on SM, and on its derivatives [14,17,19,22,23,24,25] to assign the dynamic processes to the extracted time constants reported in Table 1 and discuss the implications of our findings. This decision is supported by the similarity between the TEA spectra obtained in the current work and those reported previously for SM and its derivatives, as well as the molecular identity confirmation from mass spectrometry. Focusing initially on τ_1_, previous studies have shown that following initial photoexcitation to the 1^1^ππ* state, SM and its derivatives tend to undergo rapid geometry and vibrational relaxation out of the Franck–Condon region, along with solvent rearrangement [14,17,22]. However, we note that τ_1_ values obtained for cress sprout extract in dioxane and Detoxophane nc are within our instrument response range (~80 fs). We, therefore, attribute τ_1_ to the aforementioned dynamic processes together with coherent artefacts of the instrument response function.

The dynamic process associated with τ_2_ has been assigned to a number of different processes previously, one of which is the internal conversion (IC) of the 1^1^ππ* state to the 2^1^ππ* state via 1^1^ππ*/2^1^ππ* conical intersection (CI) [14]. However, computational studies in implicit solvent have found that only 1^1^ππ* is involved in the relaxation mechanism of SM [22,29]. As such, subsequent studies assigned this process to vibrational cooling of the 1^1^ππ* state [19]. This seems plausible given the blue shifting of EADS_3_ compared to EADS_2_ (Figure 3E,F and Figure 4C). The dynamic process associated with τ_3_ is then assigned to population flowing from the 1^1^ππ* state to the ground state, along the *trans-cis* photoisomerisation coordinate, mediated by a 1^1^ππ*/S_0_ CI. This dynamical process results in a fraction of the excited state population reforming the original *trans*-isomer, while another fraction completes the isomerisation along the allylic C=C double bond to generate the *cis*-isomer photoproduct. The *cis*-isomer photoproduct accounts for the absorption of EADS_4_ (at ~360 nm) with a time constant τ_4_, which is >2 ns [14,15,17,19,22,23,25,30].

The assignment of EADS_4_ to the *cis*-isomer is supported through a comparison of the transient absorption profile obtained at Δ*t* = 2 ns, shown as an inset in Figure 3E,F and Figure 4C, to those reported for SM and its derivatives previously [14,30], which show an identical long-lived feature assigned to the *cis*-isomer.

While the overall photodynamics of the SM within cress sprout extract and Detoxophane nc are largely complete within 100 ps, differences in the time constants and spectral profiles can be observed and warrant discussion. We briefly focus our discussion on the difference between SM in Detoxophane nc compared to SM in cress sprout extract in both dioxane and water. With regards to the time constants, those extracted from Detoxophane nc are shorter than the corresponding time constants extracted from the cress sprout extract in both dioxane and (most notably) water. The longer time constants in water are in line with previous studies of SM and its derivatives in polar solvents, compared to nonpolar counterparts [14,29]. This difference in time constants in different polarity solvents is likely due to changes in the potential energy surface of SM in the excited state; this results in a higher energy barrier to overcome along the relaxation coordinate in polar solvents. In terms of spectral profiles, in previous studies of SM and its derivatives [14,17,19,22,23,25], SE is a characteristic feature seen in the TEA spectra in polar solvents. The absence of SE in the TEA spectra of Detoxophane nc suggests that (i) the majority of the cress sprout extract is encapsulated within the lipid vesicles and (ii) the host (encapsulation) and guest (SM in the cress sprout extract) interaction within the cage is more complex than a simple interface between the SM, polar solubiliser, and hydrophilic end of the lipid vesicles. Importantly, this complex interaction between the host and guest only mildly perturbs the photodynamics of the absorbing species, as is the case in weakly perturbing non-polar solvents.

Recent toxicity studies by Peyrot et al. focused on potential endocrine disruption of SM in skincare products [31]. Their results revealed that SM possesses no endocrine disruption properties.

Taken together, the ultrafast photodynamics of SM in Detoxophane nc are not compromised by other components within the formulation. This is an important requirement of a UV filter for sunscreen formulation, enabling the UV filter to dissipate the excess energy absorbed safely and bypassing potentially harmful side reactions such as the generation of reactive oxygen species. Likewise, the ultrafast relaxation processes ensure that the UV filter is effectively recycled (i.e., retains its molecular integrity) to maintain photoprotection.

## 4. Materials and Methods

### 4.1. Steady-State Spectroscopy

All solvents used were of analytical grade unless otherwise stated.

The cress sprout extract and Detoxophane nc were studied using spectroscopy as received from “Mibelle Group Biochemistry” without further purification. For the steady-state measurement, 0.1 mg/mL of the cress sprout extract was dissolved in deionised water. The UV–Vis measurements were taken within a 1 cm path length quartz cuvette using a Cary 60 spectrometer (Agilent Technologies, Santa Clara, CA, USA). The same measurement was repeated for Detoxophane nc diluted in deionised water (1:100). SM was synthesised as described in previous studies [31,32]. For the photostability study of the cress sprout extract, the UV absorption was recorded both before irradiation and at various times during 2 h irradiation with an arc lamp (Fluorolog 3, Horiba, Jobin Yvon Inc. Kyoto, Japan). The irradiance at maximal absorption (λ_max_) of the sample was set to 200 µW, with an 8 nm full-width half-maximum (FWHM). This irradiance power is four times that of the estimated solar irradiance of the Earth’s surface (~48 µW) at the irradiation wavelength (324 nm) to accelerate any potential degradation.

^1^H NMR spectra data were collected in deuterium oxide (Sigma Aldrich, St. Louis, MO, USA) using a Bruker Avance III HD system at 400 MHz.

### 4.2. Identification of the UV-Absorbing Species

The cress sprout extract was prepared for measurement with UHPLC–HRMS for identification of the UV-absorbing species as follows. The cress sprout extract was sequentially extracted in 50% HPLC-grade methanol at 4 °C overnight followed by sonication the next day. After extraction, the solvent was pooled together, centrifuged at 4000 rpm for 30 min to get rid of debris, then the clear solvent was evaporated using the centrifugal evaporator (SP Genevac EZ-2 Series). The crude extract was then resuspended in 50:50 *v/v* water/methanol and filtered using an Amicon Ultra 3K cut-off filter for UHPLC–HRMS analysis. In a similar manner, synthesised *trans*-SM was prepared to a concentration of 20 µM in 50:50 *v/v* water/methanol as standard for UHPLC–HRMS analysis. Analysis was carried out with a 2 µL sample injection through a reverse-phase column (Zorbax Eclipse plus C18, size 2.1 × 100 mm, particle size 1.8 µm) connected to a Dionex 3000 RS UHPLC hyphenated to a Bruker Ultra High-Resolution Q TOF Maxis II mass spectrometer using electrospray ionisation (ESI) in positive mode. A *m/z* range of 50–3000 was used. A gradient elution method was programmed by increasing solvent B from 0 to 100% for 30 min (solvent A = 0.1% (*v/v*) formic acid in water; solvent B = acetonitrile with 0.1% formic acid). The system was then equilibrated for 15 min before the next injection.

### 4.3. Transient Electronic Absorption Spectroscopy (TEAS)

The femtosecond (fs) TEAS setup and procedure used to explore the photodynamics of the cress sprout extract and Detoxophane nc have been detailed previously [16,18,33,34,35], and only information specific to the present experiment is reported here. For the cress sprout extract, 0.1 g of the dried extract was dissolved in 25 mL of deionised water and separately in dioxane, whereas the Detoxophane nc was used for TEAS as received without further dilution. In all cases, the pump excitation wavelength was set at 330 nm. The sample was delivered through a demountable Harrick Scientific flow-through cell equipped with two CaF_2_ windows separated by 100 (950) µm polytetrafluoroethylene spacers for cress sprout extract (Detoxophane nc), thereby defining the optical path length of the sample. The samples were circulated using a diaphragm pump (SIMDOS, KNF) recirculating from a 25 mL sample reservoir to ensure each pump–probe pulse interacted with a fresh sample, with a maximum pump–probe delay of 2 ns.

## 5. Conclusions

In conclusion, all our studies revealed that the UV-absorbing species in cress sprout extract is mainly sinapoyl malate. Furthermore, we found through transient electronic absorption spectroscopy that the photoprotection properties of the cress sprout extract mirror that of sinapoyl malate. Finally, our studies revealed that the photoprotection properties of the sinapoyl malate in the cress sprout extract present in Detoxophane nc is not compromised by the formulation environment. While Detoxophane nc is not recommended as a substitute for organic or inorganic registered UV filters for sunscreen applications, the current study suggests that it may offer additional photoprotection to end users as a UV filter booster. However, further studies might be required to fully characterise the possible photodegradation pathways and any ensuing photoproducts together with the potential phototoxicity of SM and cress sprout extract. In addition, cell- or human-based studies may provide further validation of the photoprotection properties of garden cress sprout extract. Additionally, its possible use as a dietary supplement may require further exploration; these studies are beyond the scope of the current work.

## Figures and Tables

**Table 1:** Time constants and associated errors extracted from fitting the TEA spectra collected for cress sprout extract in water and dioxane, and Detoxophane nc.

Sample	τ_1_/fs	τ_2_/ps	τ_3_/ps	τ_4_/ns
Cress sprout extract (water)	120 ± 50	1.04 ± 0.05	17.13 ± 0.17	>2
Cress sprout extract (dioxane)	60 ± 40	0.91 ± 0.04	14.84 ± 0.35	>2
SM (water)	100 ± 50	0.88 ± 0.05	15.50 ± 0.06	>2
Detoxophane nc	60 ± 40	0.52 ± 0.04	11.90 ± 0.20	>2

## Data Availability

The datasets presented in this study can be found in online repositories. The names of the repository/repositories and accension number can be found below: Zenodo repository DOI: 10.5281/zenodo.5777202.

## Supplementary Materials

The following are available online: Figures S1 and S2: ^1^H NMR spectrum (400 MHz) of synthesized *trans*-sinapoyl malate and cress extract in D_2_O. Figure S3: Photostability of garden cress. Figures S4 and S5: Before and After Irradiation ^1^H NMR spectrum of SM and garden cress extract. Figure S6: UV chromatogram of SM and garden cress extract. Figure S7: UV spectrum of garden cress extract from UHPLC elution. Figure S8: TEAS of SM in water. Figure S9: Residuals for the sequential fit to the TEA spectra. Figure S10: Instrument response function.

## Sample Availability

Samples of the cress sprout extract and Detoxophane nc are available from the authors.
