# Supplementary material for: Unravelling the Photoprotection Properties of Garden Cress Sprout Extract
Source: Molecules. 2021 Dec 16;26(24):7631. doi: 10.3390/molecules26247631 (PMC8705737; doi:10.3390/molecules26247631)
Supplement: Supplementary file 1 [file molecules-26-07631-s001.zip › molecules-1479400-supplementary.pdf]

molecules

MDPI

Article

Supporting Information

Unravelling the Photoprotection Properties of Garden Cress Sprout Extract

Temitope T. Abiola [1], Nazia Auckloo [1,2], Jack M. Woolley [1], Christophe Corre [1,2], Stéphane Poigny [3], Vasilios G. Stavros [1,*]

[1] Department of Chemistry, University of Warwick, Coventry, CV4 7AL, UK; temitope.abiola@warwick.ac.uk (T.T.A.); Nazia.Auckloo@warwick.ac.uk (N.A.) jack.woolley@warwick.ac.uk (J.M.W.); C.Corre@warwick.ac.uk (C.C.)
[2] Warwick Integrative Synthetic Biology Centre and School of Life Sciences, University of Warwick, Coventry, CV4 7AL, UK
[3] Mibelle Group Biochemistry, Mibelle AG, Bolimattstrasse 1, CH-5033 Buchs, Switzerland; stephane.poigny@mibellegroup.com (S.P.)
* Correspondence: v.stavros@warwick.ac.uk (V.G.S.)

1. Sinapoyl malate and garden cress sprout extract NMR analysis

The ^{1}H NMR spectra of synthesized *trans*-sinapoyl malate (SM) and garden cress sprout extract obtained in deuterium oxide (D_2O, Sigma Aldrich) are reported in Figure S1 and S2, respectively. The data revealed that the absorbing species in garden cress sprout extract have identical spectra with synthesized *trans*-SM. We note here that the spectrum of garden cress sprout extract from 4.5 – 0 ppm is obscured with other plant molecular component, hence we refrain from the assignment of the peaks in this region.

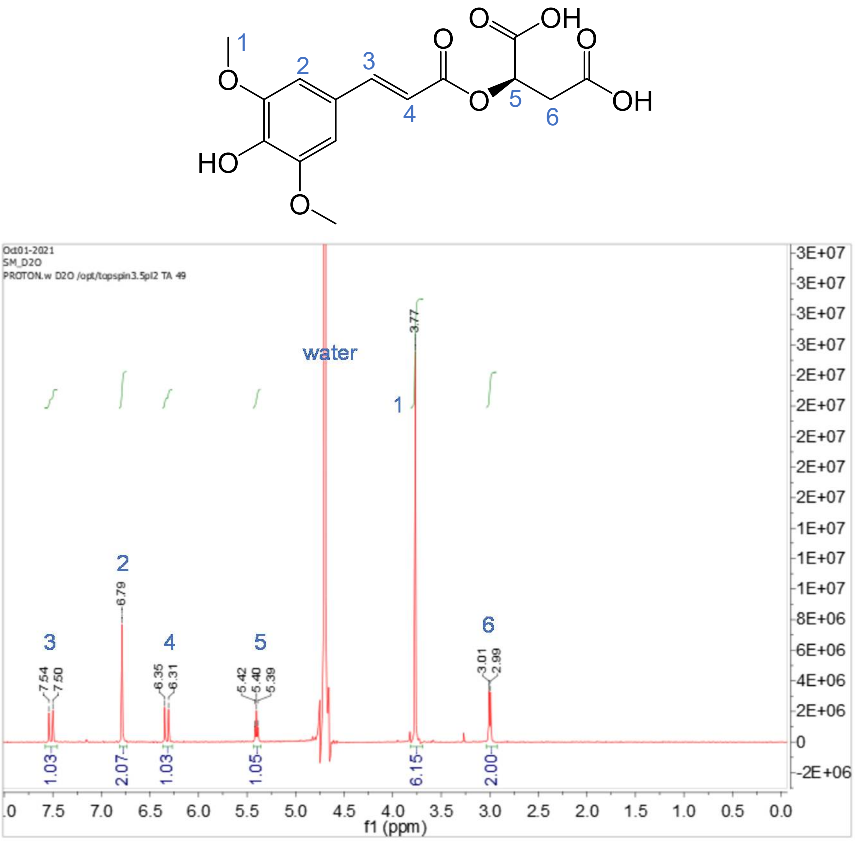

Figure S1. ^{1}H NMR spectrum (400 MHz) of synthesized *trans*-sinapoyl malate in D_2O.

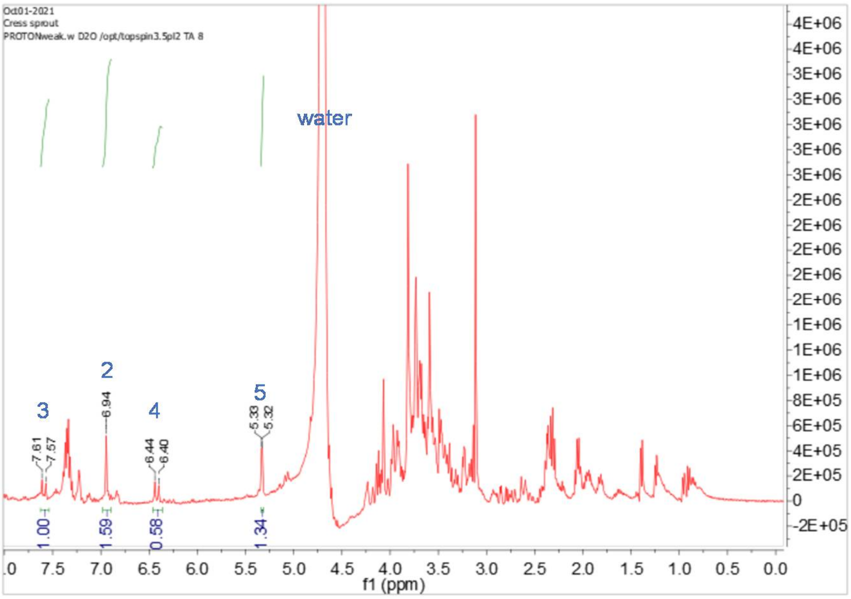

Figure S2. ^{1}H NMR spectrum (400 MHz) of garden cress sprout extract in D_2O.

2. Long-term photostability test of garden cress sprout extract

The result of the photostability test on garden cress sprout extract is reported in Figure S3. This result showed impressive photostability with 18% degradation at photostationary state, similar to results reported for sinapoyl malate previously [1]. The reduction in absorbance is likely due to the formation of the *cis*-isomer having lower extinction coefficient. However, further studies should be conducted with high-resolution mass spectrometry to determine all the possible photofragments that may be formed together with the *cis*-isomer following irradiation.

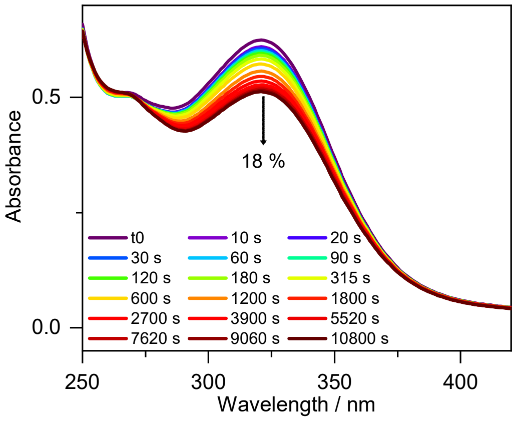

Figure S3. Long-term photostability of garden cress sprout extract in water. The UV-visible spectrum of the sample obtained at varying duration of irradiation with a xenon arc lamp. The downward arrow denotes the observed decrease at the peak absorbance over 120 min of irradiation. The absorbing species achieved photostationary state within this irradiation time.

In order to validate the formation of *cis*-isomer of the absorbing species following irradiation, we have obtained and reported the ^{1}H NMR of the SM present in garden cress sprout extract before and after UV light irradiation and compared to those of synthetic *trans*-SM before and after irradiation. These data are presented in Figure S4 and S5. The irradiation was carried out for 2 hr in a 1mm pathlength cuvette with an arc lamp (Fluorolog 3, Horiba). The irradiance at maximal absorption (λ_{max}) at the sample was set to 400 µW and 2 mW for SM and garden cress sprout extract respectively, with an 8 nm full width half maximum (FWHM). The concentration of the samples were: (1) 0.7 mg/mL for SM; and (2) 10 mg/mL for garden cress sprout extract. The higher concentration of the latter is due to the other plant-based components a solution.

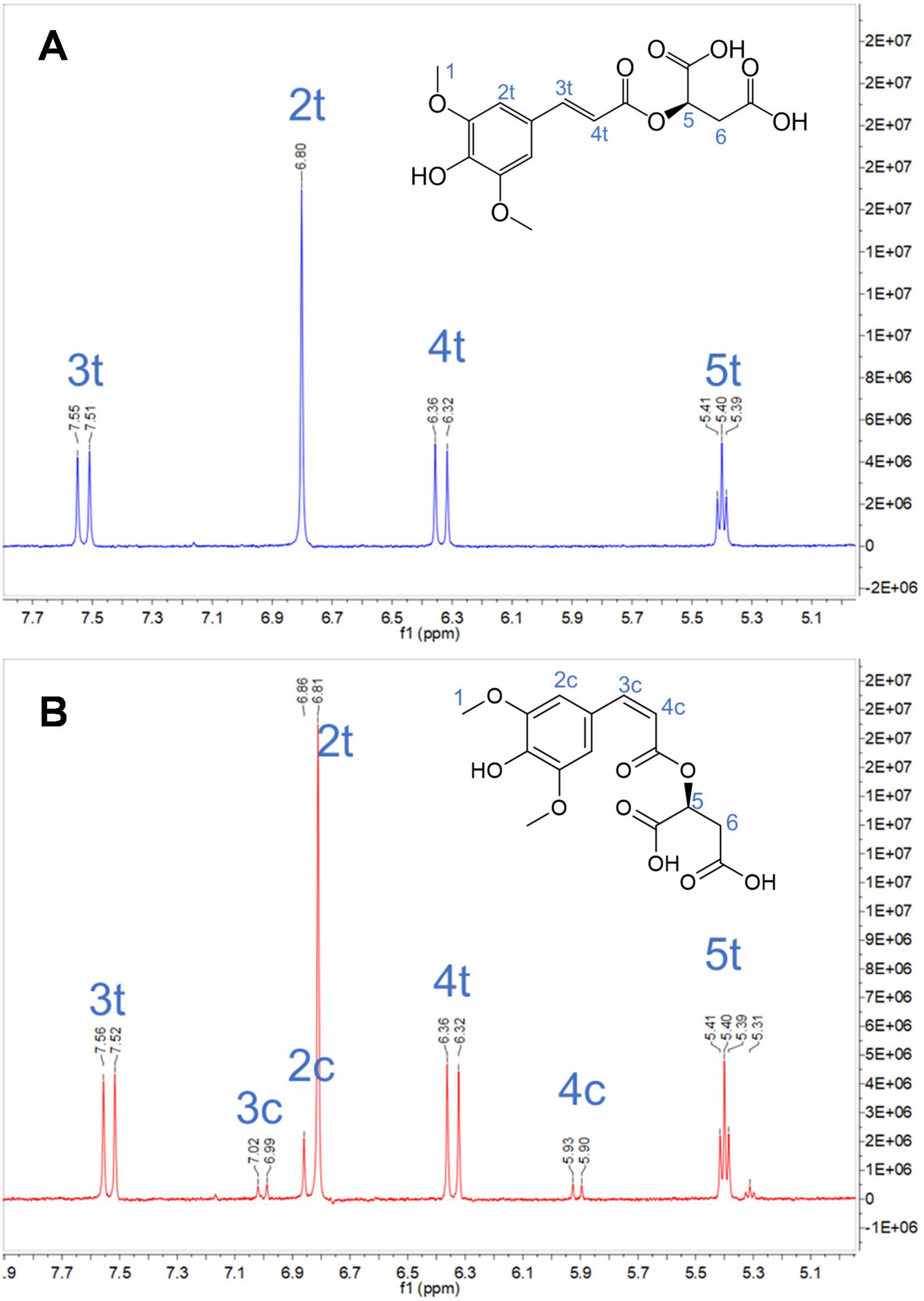

Figure S4. ^{1}H NMR spectrum (400 MHz) of synthesized *trans*-sinapoyl malate in D_2O (A) before irradiation, revealing only the proton peaks from *trans*-isomer, and (B) after irradiation, revealing proton peaks for both *trans*- and *cis*-isomer. The "t" and "c" in the NMR peak annotation denote *trans* and *cis* respectively. The coupling constant (calculated by multiplying the difference between the doublet peaks in ppm by the frequency of the NMR used, 400 MHz) for H-3t/H4t and H-3c/H4c are 16 and 12 Hz respectively and consistent with those previously reported for SM derivatives [2]. This strongly supports photoisomerization occurring during irradiation leading to the formation of *cis*-isomer photoproduct. As a visual aid, we have shown only the region where isomerization peaks are observed *i.e.*, 7.9 – 5 ppm, full NMR spectrum is reported in Figure S1.

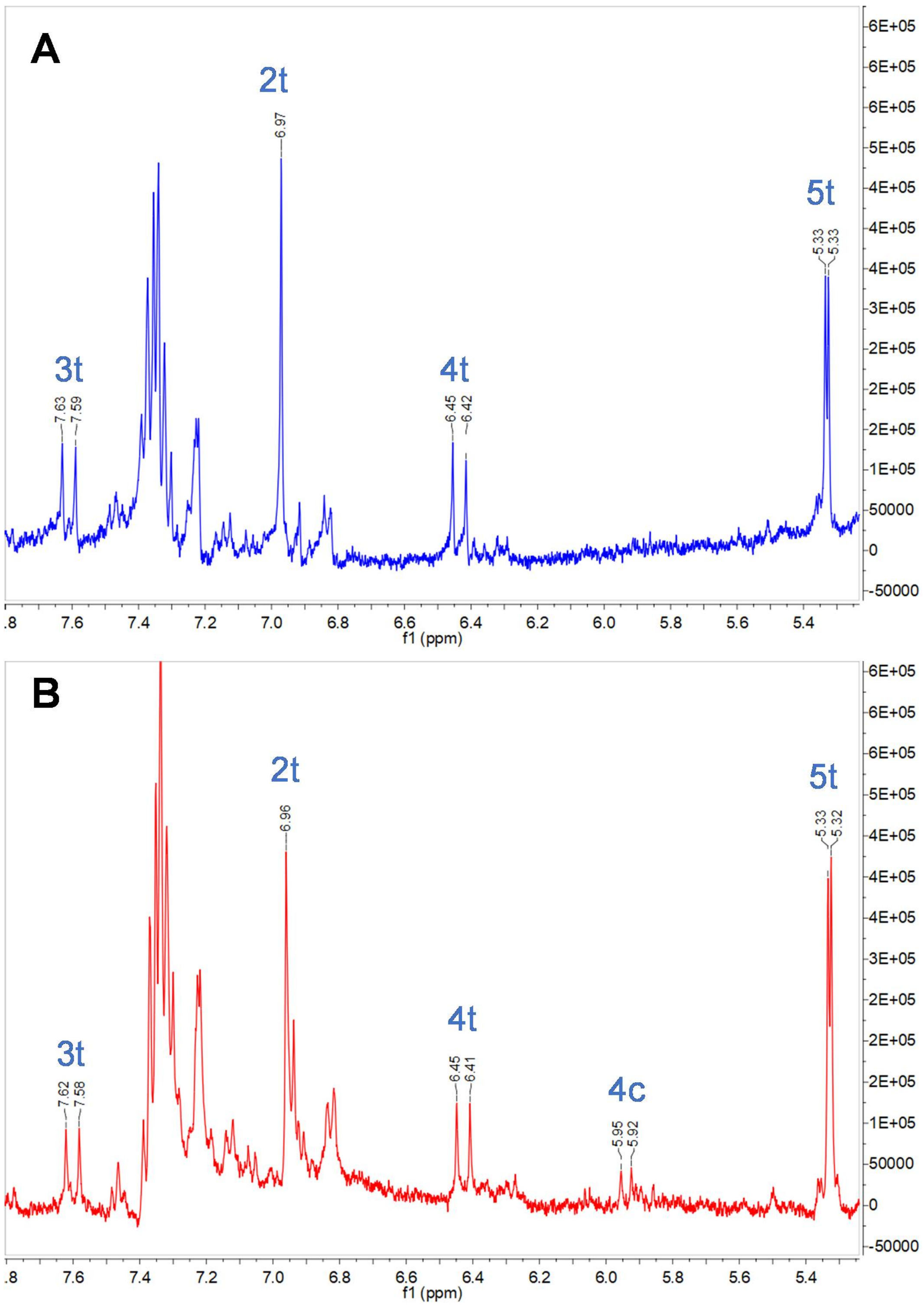

Figure S5. ^{1}H NMR spectra (400 MHz) of garden cress sprout extract in D_2O (A) before irradiation, revealing only the proton peaks from *trans*-isomer, and (B) after irradiation revealing proton peaks for both *trans*- and *cis*-isomer. The "t" and "c" in the NMR peak annotation denote *trans* and *cis* respectively. The coupling constant (calculated by multiplying the difference between the doublet peaks in ppm by the frequency of the NMR used, 400 MHz) for H-3t/H4t and H4c are 16 and 12 Hz respectively and consistent with those previously reported for SM derivatives[2]. This strongly supports photoisomerization occurring during irradiation leading to the formation of *cis*-isomer photoproduct. As a visual aid, we have shown only the region where isomerization peaks are observed *i.e.*, 7.9 – 5 ppm, full NMR spectrum is reported in Figure S2. Assignment of the peaks compares well with those from synthetic SM. We note the absence of the 3c and 2c proton peaks following irradiation, which are likely buried under the intense proton peaks from other components in the cress extract.

3. Additional UHPLC-HRMS data.

In addition to the UHPLC-HRMS data reported in the manuscript, additional data are reported herein to support the interpretation and identification of the absorbing specie in garden cress sprout extract. The UV chromatogram reported in Figure S6 revealed the same elution time for UV absorbing specie in garden cress sprout extract and sinapoyl malate standard. In the bottom panel, the mixture of garden cress sprout extract and sinapoyl malate standard co-eluted together. These further validate the assignment of the absorbing species as sinapoyl malate. Also, we reported in Figure S7 the UV absorption profile of the garden cress sprout extract eluted from the UHPLC at 15.8-15.9 min; the resulting spectrum is near-identical to the UV profile of sinapoyl malate. Taken together these experimental data, we conclude that the absorbing species is sinapoyl malate.

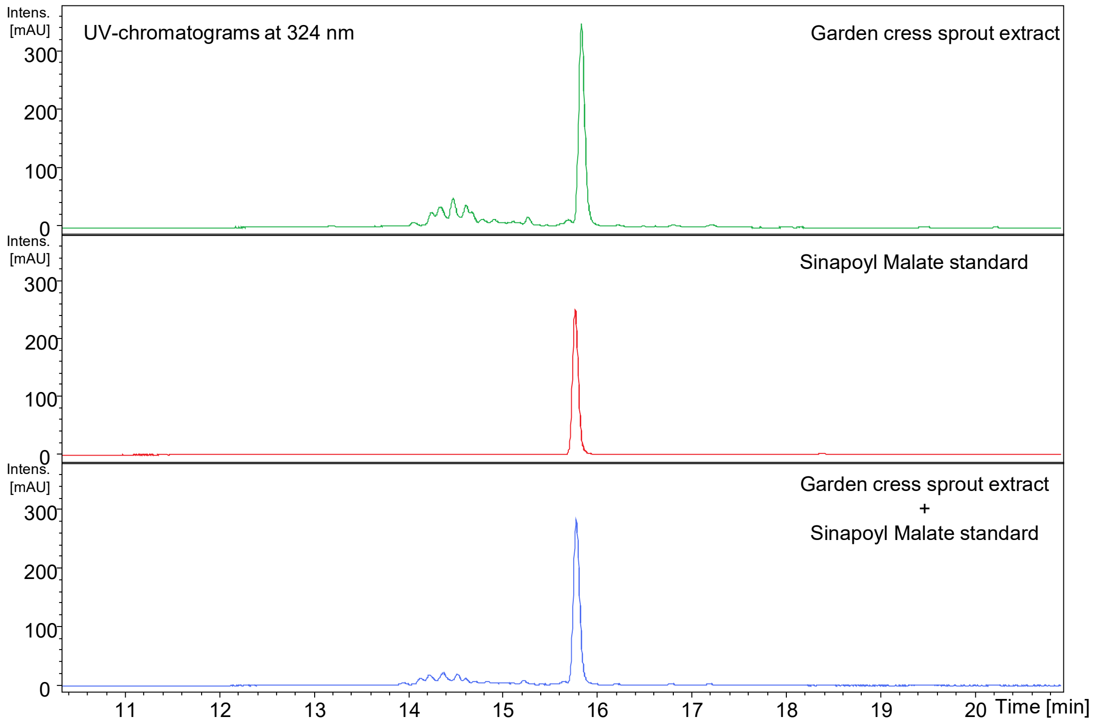

Figure S6. UV chromatogram obtained in 50:50 v/v water: methanol at 324 nm for garden cress sprout extract (top panel, green), sinapoyl malate standard (middle panel, red) and a mixture of sample and standard (bottom panel, blue). The results indicate the same elution time from the chromatogram for all the data.

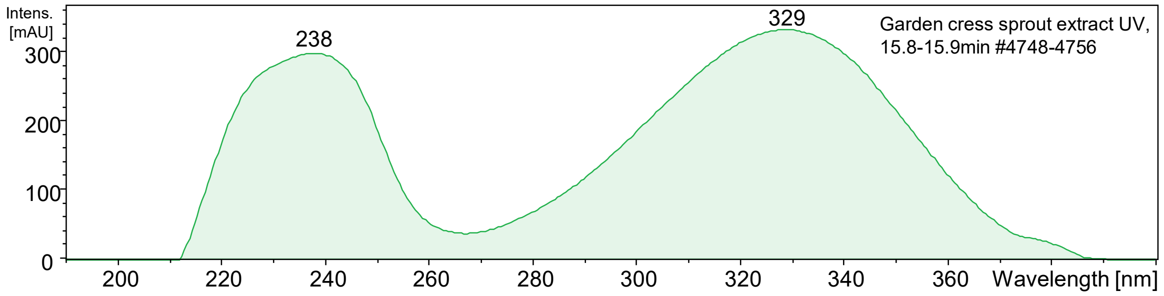

Figure S7. UV spectrum of the garden cress sprout extract elution from the UHPLC at 15.8 – 15.9 min in a solution of 50:50 v/v water: methanol.

4. TEA spectra of SM in water

The TEA spectra together with the EADS obtained from SM in water are reported in Figure S8. The data, and time constants reported in Table 1 (see manuscript) are comparable to the data obtained for cress sprout extract in water. These similarities further confirm that SM is present in garden cress sprout extract.

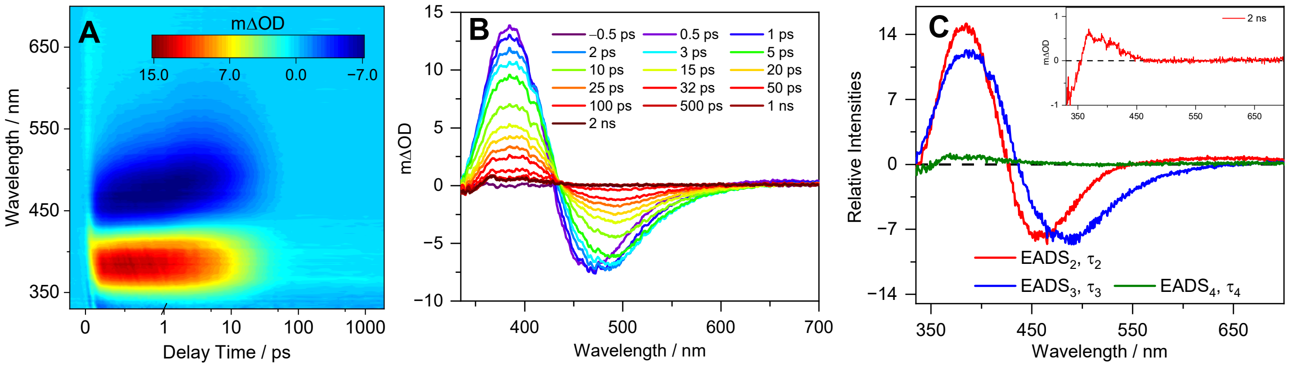

Figure S8. TEA spectra obtained for the bulk solution of SM in water photoexcited at 322 nm shown as a false colour map (A). The same data is presented as a line plot of mΔOD vs probe wavelength at selected pump-probe delay times in (B). The EADS is shown in (C) with the 2 ns transients presented as inset.

5. Residuals for the Sequential Fit to the TEA Spectra

The residuals from the sequential global fitting with respect to the raw TEA spectra data (*i.e.*, the difference between the fit and the raw data at each data point) are shown in Figure S9. The small-signal intensities of the residual compared to the raw TEA spectra demonstrate the quality of the fits.

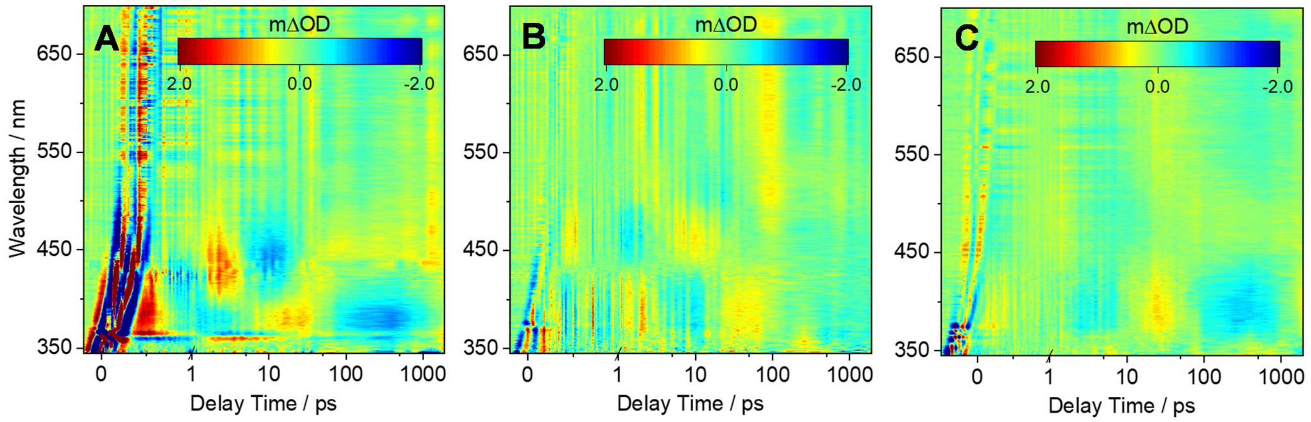

Figure S9. False colour maps of the residuals of the fit for the TEA spectra of garden cress sprout extract in (A) dioxane (B) water and (C) Detoxophane nc.

6. Instrument Response Function

The TEAS measurements of the time zero solvent-only scan were recorded to obtain the instrument response function (IRF), which determines the limiting temporal resolution of the present experiments. Water showed a very weak time-zero response following excitation at 330 nm with pump-pulse power of 500 μW (employed for the experiment). This implies that there are no (appreciable) solvent dynamics in our data. Nonetheless, we have taken the solvent response for water at a higher pump-pulse power (1 mW) to account for the temporal resolution of our experiment in water. Furthermore, we note that our instrument response function in water has a strong contribution of cross-phase modulation between pump and probe pulses as they traverse our solvent medium. However, this should not affect the conclusions of the manuscript given the longer timescales we are investigating in the solute. For this reason, we chose to follow the approach of Kovalenko *et al.* [3], in which we use a frequency-dependent cross-correlation function to model our solvent response. The value obtained for the temporal resolution of the solvent-only time zero response shown in Figure S10 are 80 and 100 fs for dioxane and water, respectively.

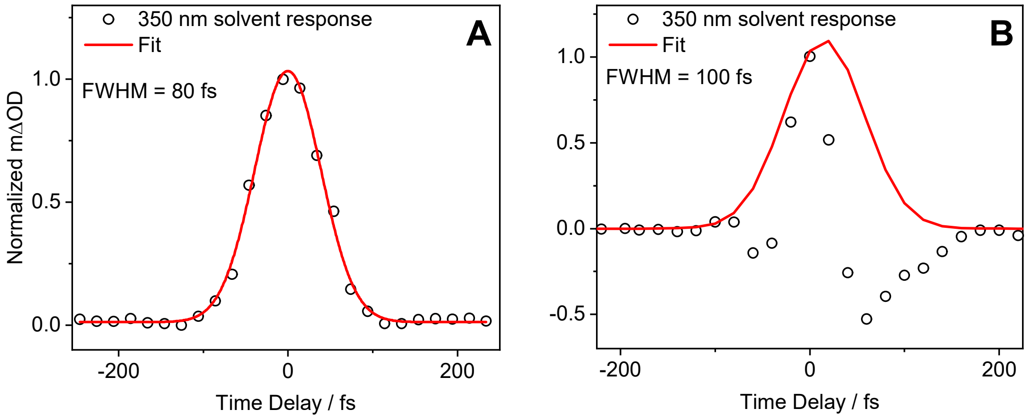

Figure S10. Solvent-only time-zero response at a probe wavelength of 350 nm following photoexcitation at 330 nm for (A) dioxane and (B) water. The excitation wavelengths for the solvents presented herein correspond to the wavelength at which the samples were excited in each solvent. The extracted full-width half maxima are shown in each panel. These values are used as the instrument response in the corresponding global fit analysis of TEA spectra.

References

1. Peyrot, C., et al., *Expeditious and sustainable two-step synthesis of sinapoyl-l-malate and analogues: towards non-endocrine disruptive bio-based and water-soluble bioactive compounds.* Green Chem., 2020. **22**(19): p. 6510-6518.
2. Horbury, M.D., et al., *Investigating isomer specific photoprotection in a model plant sunscreen.* Chem. Commun., 2018. **54**(8): p. 936-939.
3. Kovalenko, S.A., et al., *Femtosecond spectroscopy of condensed phases with chirped supercontinuum probing.* Phys. Rev. A., 1999. **59**(3): p. 2369.
